# Zinc ion thermal charging cell for low-grade heat conversion and energy storage

**DOI:** 10.1038/s41467-021-27755-x

**Published:** 2022-01-10

**Authors:** Zhiwei Li, Yinghong Xu, Langyuan Wu, Yufeng An, Yao Sun, Tingting Meng, Hui Dou, Yimin Xuan, Xiaogang Zhang

**Affiliations:** 1 Jiangsu Key Laboratory of Electrochemical Energy Storage Technologies, College of Material Science and Engineering, Nanjing University of Aeronautics and Astronautics, 211106 Nanjing, China; 2 School of Energy and Power Engineering, Nanjing University of Aeronautics and Astronautics, 210016 Nanjing, P. R. China

**Keywords:** Batteries, Materials for energy and catalysis

## Abstract

Converting low-grade heat from environment into electricity shows great sustainability for mitigating the energy crisis and adjusting energy configurations. However, thermally rechargeable devices typically suffer from poor conversion efficiency when a semiconductor is employed. Breaking the convention of thermoelectric systems, we propose and demonstrate a new zinc ion thermal charging cell to generate electricity from low-grade heat via the thermo-extraction/insertion and thermodiffusion processes of insertion-type cathode (VO_2_-PC) and stripping/plating behaviour of Zn anode. Based on this strategy, an impressively high thermopower of ~12.5 mV K^−1^ and an excellent output power of 1.2 mW can be obtained. In addition, a high heat-to-current conversion efficiency of 0.95% (7.25% of Carnot efficiency) is achieved with a temperature difference of 45 K. This work, which demonstrates extraordinary energy conversion efficiency and adequate energy storage, will pave the way towards the construction of thermoelectric setups with attractive properties for high value-added utilization of low-grade heat.

## Abstract (web summary)

Low-grade heat conversion has recently emerged and displayed great promise in sustainable electronics and energy areas. Here, the authors propose a new zinc ion thermal charging cell with hybrid behaviours for high value-added conversion from heat to electricity.

## Introduction

The high value-added utilization of plentiful and sustainable heat power has spurred urgent development of cost-effective and safe technologies for harvesting low-grade heat (<100 °C) into electricity^1–3^. Among various advanced systems, electronic thermoelectric devices (e-TEs) using narrow-bandgap semiconductors can realize the conversion of low-grade heat to meet the needs of the electronic market (i.e., low-cost, safety, high efficiency) based on the Seebeck effect. For a typical e-TE material, the Seebeck coefficient (*α*) is only ~100 µV K^−1^^4^. As a result, it is very challenging to generate a sufficient voltage from 1 to 5 V by the integration of numerous e-TEs. Therefore, a new type thermal charging cell with enhanced performance has to be further designed and considered.

Recently, an alternative approach, namely, ionic thermoelectric devices (i-TEs), was adopted for direct energy harvesting, which delivers two different mechanisms including thermogalvanic effect and thermodiffusion effect^2,5^. Low-grade heat can be continuously converted into electricity because a temperature difference can induce a voltage difference. For instance, a thermally chargeable supercapacitor was demonstrated by Zhang and co-workers based on the thermodiffusion effect. An *α* value of 1.21 mV K^−1^ could be obtained at a temperature difference of 52 K with 1.0 mol L^−1^ KNO_3_ electrolyte and a porous carbon (PC) electrode^6^. However, the heat-to-current performance in this thermally chargeable supercapacitor is still limited by the adsorption/desorption and diffusion of electrolyte ions. An alternative strategy is to design an electrolyte by introducing redox couples such as ferro/ferricyanide [Fe(CN)_6_^4–^/Fe(CN)_6_^3–^]^2,5,7–10^. Liu et al. has reported ionic thermoelectric materials using KCl and [Fe(CN)_6_^4–^/Fe(CN)_6_^3–^] for the synergistic effect of thermogalvanic and thermodiffusion^2^. Consequently, a superior *α* value of 17.0 mV K^−1^ was achieved using body heat and an output voltage of over 2 V was generated by integrating 25 unipolar units. Among various thermal systems, liquid-state thermocells show great advantages in terms of their cost-effectiveness and scalability for low-grade heat conversion. Although considerable effort has been devoted to realizing a highly efficient conversion of low-grade heat, the output voltage is greatly limited by the electrode/electrolyte interface and energy storage mechanism. In addition, the low Carnot-relative efficiency (<5%) has been challenging to improve even under ideal laboratory conditions. It is reasonable to propose that the construction of a hybrid device based on the Soret effect may exhibit high potential in the harvesting of electricity with a steady temperature gradient.

**Fig. 1:**
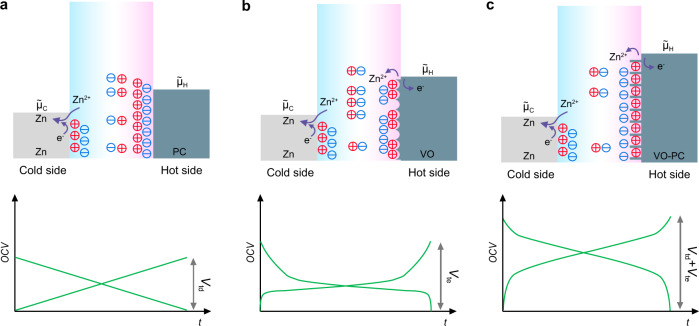
Concept and working mechanism of Zn-based thermally chargeable systems. **a** PC-based thermodiffusion effect, **b** VO-based thermoextraction effect, and **c** VO-PC-based synergistic effect.

Aqueous zinc ion batteries (ZIBs) have emerged as one of promising candidates for energy storage due to the merits of Zn anodes, such as cost-effectiveness, multivalent feature, and satisfactory stability^11–14^. When introducing the concept of ZIBs into heat-to-electricity conversion, various mechanisms can be proposed through the synergistic effect among the thermogalvanic effect of Zn anodes and the thermodiffusion and thermoextraction of ions in electrolytes and cathodes, respectively (Fig. 1). The Zn-based thermally chargeable supercapacitors using capacitive cathodes (i.e., PC) can generate electricity based on the thermodiffusion effect of electrolyte ions along with the stripping and plating of Zn anode. Typically, the applied temperature gradients can cause both cations and anions to migrate from the hot side to the cold side (Fig. 1a). A thermodiffusive voltage can be generated and defined as Δ*V*_td_ = −(û_H_ − û_C_)/*e*. In general, the intercalated Zn^2+^ migrates to the surface of Zn anode and spontaneously experiences a plating process. Based on the extraction of ions inserted in the selected cathode (VO), the transferred charges to the hot side reasonably improve the obtained electrochemical potential (Fig. 1b). However, the relatively sluggish kinetics of pure battery-like systems greatly impede their practical applications. Therefore, the Zn-based thermal charging cells (ZTCCs), which combine the thermodiffusion of ions on PC and the extraction of ions from an insertion-type VO electrode, can output a higher voltage (Fig. 1c). Notably, vanadium-based oxides demonstrate promising performances owing to their multiple valence feature and unique crystal structure for ion storage^11,13,15^. However, the sluggish kinetics, poor conductivity, and structural deterioration involved in the insertion/extraction of Zn^2+^ always hinder their large-scale applications, that is, the rational design of satisfying vanadium-based oxides for energy storage and conversion is still in its infancy.

Herein, we propose and develop promising ZTCCs by combining Zn anode and VO_2_-PC cathode. As a result, the VO_2_-PC-based ZTCC can generate electricity from low-grade heat through the synergistic effect between thermodiffusion and thermoextraction. Remarkably, a high Seebeck coefficient of 12.5 mV K^−1^ and conversion efficiency of 0.95% (7.25% of Carnot-relative efficiency) can be attained with a temperature gradient of 45 K. Moreover, VO_2_-PC shows promising capacity, good rate capability, and excellent durability for energy storage. In addition, the proof-of-concept set-up displays satisfying application potential for harvesting energy from waste heat due to its ultrahigh output voltage of ~1 V with only one unit.

## Results

### Preparation and characterization of VO_2_-PC nanosphere

The spherical vanadium-polydopamine (V-PDA) composites were rationally synthesized by a solution-based strategy using dopamine and ammonium metavanadate as carbon source and vanadium source, respectively. After pyrolysis under argon atmosphere, the PDA and V-based species were evolved into hierarchical PC matrix and anchored vanadium dioxide (VO_2_) along with gas escape, and the details can be seen in the “Methods” section. Field-emission scanning electron microscope (SEM) and transmission electron microscope (TEM) were employed to observe the microstructures of as-prepared materials. It can be found that the morphology of pure PDA-derived PC is uneven nanospheres with different diameters ranging from 100 nm to ~1 μm (Supplementary Fig. 1a, b). In particular, the VO_2_-PC is engineered by uniform nanospheres with a size of about 250 nm (Supplementary Fig. 2a, b). Significantly, the VO_2_ is uniformly distributed in the VO_2_-PC sample with a high content of 65.4 wt% determined by the thermogravimetric analyses (Supplementary Fig. 3). Through the obvious spherical morphology (Supplementary Fig. 2c), it is can be noted that the porous VO_2_-PC shows an amorphous feature. From the high-resolution TEM image of VO_2_-PC (inset of Supplementary Fig. 2c), a lattice fringe with the spacing of 0.353 nm can be attributed to the (110) plane of monoclinic VO_2_. Such unique integration of VO_2_ and PC in the VO_2_-PC would be beneficial to provide abundant pathways and electroactive sites for Zn^2+^ diffusion and storage, which could enhance the rate capability and specific capacity of as-designed devices. The energy-dispersive X-ray spectrometry elements mapping images in Supplementary Fig. 2d further confirm the coexistence and extremely even distribution of C, N, O, and V elements in the VO_2_-PC sphere. The X-ray diffraction (XRD) pattern of VO_2_-PC is displayed in Supplementary Fig. 2e, in which almost all the peaks can be well indexed to the monoclinic VO_2_ (PDF#81-2392)^16^. The crystal structure of layered VO_2_ is presented in the inset of Fig. 1e, and relatively large one-dimensional tunnels are formed by the shared corners of V_4_O_10_ in VO_2_, implying fast transport of electrolyte ions. Supplementary Fig. 2f shows the Raman spectrum of VO_2_-PC. The characteristic Raman shifts can be assigned to the layered structure of VO_2_ crystalline (140.2 cm^−1^), V=O bending vibration (279.4 and 406.7 cm^−1^), V-O-V stretching vibration (686.9 cm^−1^), and V=O stretching vibration (992.6 cm^−1^)^17^. Besides, the valence of V element was investigated by the X-ray photoelectron spectroscopy (XPS) spectrum in Supplementary Fig. 2f. As analyzed, only V^4+^ exists in the obtained product, which suggests the successful preparation of standard VO_2_^18^. More importantly, when using molybdenum (Mo) and tungsten (W) as the metal sources, this proposed strategy also can reasonably form the corresponding Mo_4_O_8_-PC and W_3_O_9_-PC, as proved by the SEM images and XRD results (Supplementary Fig. 4).

### Electrochemical performance of ZTCCs

**Fig. 2:**
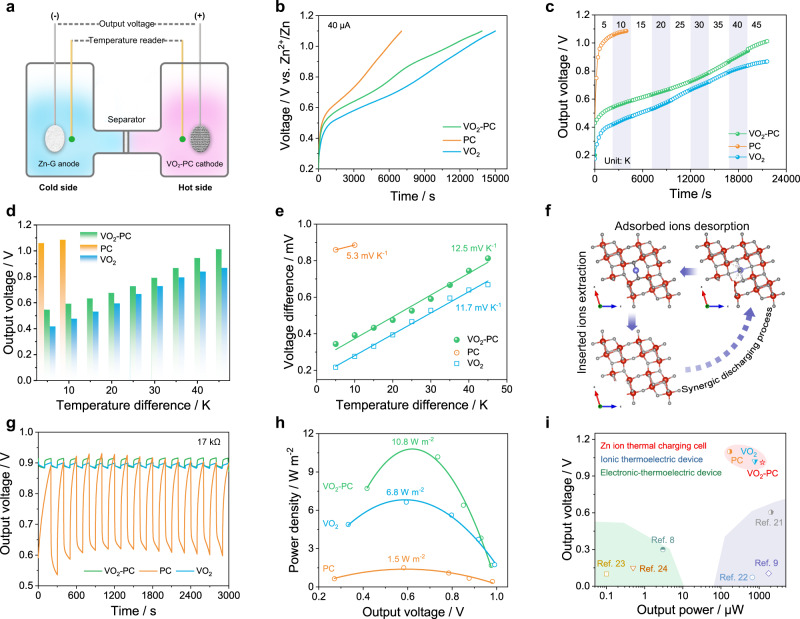
Construction and performance of ZTCCs. **a** The schematic diagram of the non-isothermal cell. **b** Electrochemical charge curves with a current of 40 μA. **c** Evolution of the output voltage with various temperature gradients. **d** The output voltage at various temperature differences. **e** Voltage difference vs. temperature difference. **f** The corresponding structural changes of VO_2_ during energy conversion. **g** The discharging curves with a load of 17 kΩ. **h** Plots of current densities and power densities under various output voltages. **i** Comparison in output power and voltage.

To accurately evaluate the practical application of electrode materials in the conversion of low-grade heat, we have constructed a non-isothermal H cell with Zn-G anode and VO_2_-PC cathode, as illustrated in Fig. 2a. During the measurements, the VO_2_-PC cathode pre-intercalated with Zn^2+^ is heated by hot water bath while the Zn-G anode is unheated to form a temperature difference (Δ*T*). It should be noted that the Δ*T* between anode and cathode is detected by the thermocouple inserted in each chamber. To minimize the kinetics effects, an ultralow current of 40 μA was applied to various electrodes without temperature gradient (Fig. 2b). Clearly, the VO_2_-PC combines the characteristics of its components including high porosity of PC and electrical activity of VO_2_, which endows VO_2_-PC electrode with a shorter time to achieve the open-circuit voltage than pure VO_2_ under this case. The fast increase of voltage at the initial state is mainly caused by the desorption of electrolyte ions. Meanwhile, the platforms around 0.6 and 0.9 V that appeared in both VO_2_ and VO_2_-PC are assigned to the extraction of ions. The evolution of the output voltage (Δ*V*) under various temperature differences for the VO_2_-PC-, VO_2_- and PC-based ZTCCs are profiled in Fig. 2c. As we gradually increase the heat input, the temperature difference and output voltage can achieve a series of steady states within 2400 s. Notably, the output voltage is dramatically increased with a Δ*T* of 5 K for PC-based ZTCC, which is mainly caused by the fast desorption of electrolyte ions. When the Δ*T* reaches 10 K, a relatively stable voltage of ~1.1 V can be obtained. Interestingly, the output voltage caused by the desorption of electrolyte ions in the initial state (Δ*T* = 5 K) is relatively weakened and only ~0.5 V can be achieved for VO_2_-PC, which is also higher than that of VO_2_ (~0.4 V). This significant difference between PC and VO_2_-PC may be highly determined by their different charge storage mechanisms. As discussed above, the inserted Zn^2+^ can react with the VO_2_ and achieve stable bonding in the form of chemical bonds^19^. Thus, great heat is required to release such combined Zn^2+^, which also implies a high energy input. Consequently, a comparable output voltage of ~1.01 V can be delivered from the VO_2_-PC-based ZTCC under the temperature difference of 45 K. However, only ~0.87 V is achieved for VO_2_-based set-up due to the sluggish extraction of ions from the highly ordered crystals. Meanwhile, we have performed XRD patterns of electrodes after the thermal charging process from 5 to 45 K, as shown in Supplementary Fig. 5. The (110) plane of VO_2_ shifts from 25.8° to ~25° due to the Zn^2+^ insertion during the electrochemical discharging process (from the initial state to 5 K-s). The VO_2_-PC cathode remains unchanged at the temperature difference of 5 K (from 5 K-s to 5 K-e), but the peaks are changed and slightly shifted from ~25° to 25.3° during 10 to 45 K. This interesting phenomenon suggests that the rapid desorption of adsorbed ions on the VO_2_-PC does not change the electrode structure. However, the crystal structure of VO_2_ can be partially changed by gradual extraction of inserted ions under temperature gradient, which is mainly induced by the redox reaction between Zn_*x*_VO_2_ and VO_2_. These findings also are in line with the electrochemical results. Figure 2d summarizes the detailed output voltage value at various temperature differences of as-constructed ZTCCs. Obviously, the increasing trend of Δ*V* is highly consistent with the kinetics of adsorption/desorption and insertion/extraction. According to the relationship between Δ*V* and Δ*T*, the temperature/Seebeck coefficient (*α*) can be calculated, which is described by the following equation^6,20^:

1 $$\alpha = \frac{\partial V}{\partial T}$$

As shown in Fig. 2e, an improved thermopower from 5.3 to 12.5 mV K^−1^ can be achieved by introducing VO_2_ into PC matrix using an aqueous electrolyte. Compared with the PC and pure VO_2_, the higher *α* value of VO_2_-PC is probably caused by the synergistic effect of thermodiffusion and thermoextraction. For convenience, the optimized crystal structures of VO_2_, Zn-VO_2_, and Zn/H_2_O-VO_2_ are displayed in Fig. 2f. The possible electrochemical processes involved in both electrodes can be described as follows:

Cathode:

2 $${{{{{{\rm{VO}}}}}}}_{2}+{x{{{{{\rm{Zn}}}}}}}^{2+}+{y{{{{{\rm{H}}}}}}}_{2}{{{{{\rm{O}}}}}}+2x{{{{{{\rm{e}}}}}}}^{-}\leftrightarrow {{{{{{\rm{Zn}}}}}}}_{x}{{{{{{\rm{VO}}}}}}}_{2}\cdot y{{{{{{\rm{H}}}}}}}_{2}{{{{{\rm{O}}}}}}$$

Anode:

3 $$x{{{{{\rm{Zn}}}}}}\leftrightarrow x{{{{{{\rm{Zn}}}}}}}^{2+}+2x{{{{{{\rm{e}}}}}}}^{-}$$

With the change of Δ*T*, the reversible “breathing” of VO_2_ unit cell together with the insertion/extraction of electrolyte ions can realize the continuous conversion and utilization of low-grade heat. It is worth mentioning that the relative contribution to the total thermopower can be distinguished as follows: 3.6% of redox entropy of Zn/Zn^2+^, 30.1% of redox entropy of Zn_*x*_VO_2_/VO_2_, 7.6% contribution of thermodiffusion of Zn(CF_3_SO_3_)_2_ electrolyte, and 58.7% contribution of thermodiffusion of electrolyte ions in VO_2_-PC electrode (Supplementary Note 1 and Supplementary Fig. 6). Motivated by the high voltage of as-assembled ZTCCs, a fixed resistor (17 kΩ) was employed to study the corresponding rechargeable behavior. Significantly, the energy decay and thermocharging behavior of VO_2_-PC-based ZTCC is much comparable to that of PC- and VO_2_-based ZTCC even over 3000 s (15 cycles) with a stable voltage of 0.9 V, demonstrating the satisfying performance of VO_2_-PC-based ZTCC in electronics (Fig. 2g). In addition, the relatively rapid chargeability of ZTCCs within 100 s can be highly determined by their low energy decay and fast charge response. Furthermore, a series of fixed resistors were used to investigate the power density (*P*) according to *P* = *V*^2^/*R*. More excitingly, an ultrahigh power density of 10.8 W m^−2^ can be fitted by VO_2_-PC-based ZTCC with a load resistor of 200–470 Ω, which is much better than that of PC- and VO_2_-based ZTCCs (Fig. 2h). When comparing the output voltage and power of ZTCCs with other reported systems, as-built ZTCCs are more competitive than that of previously reported electronic–thermoelectric devices and ionic thermoelectric devices (Fig. 2i), demonstrating the availability of ZTCCs as promising devices for low-grade heat conversion^8,9,21–24^.

### Conversion efficiency of ZTCCs

**Fig. 3:**
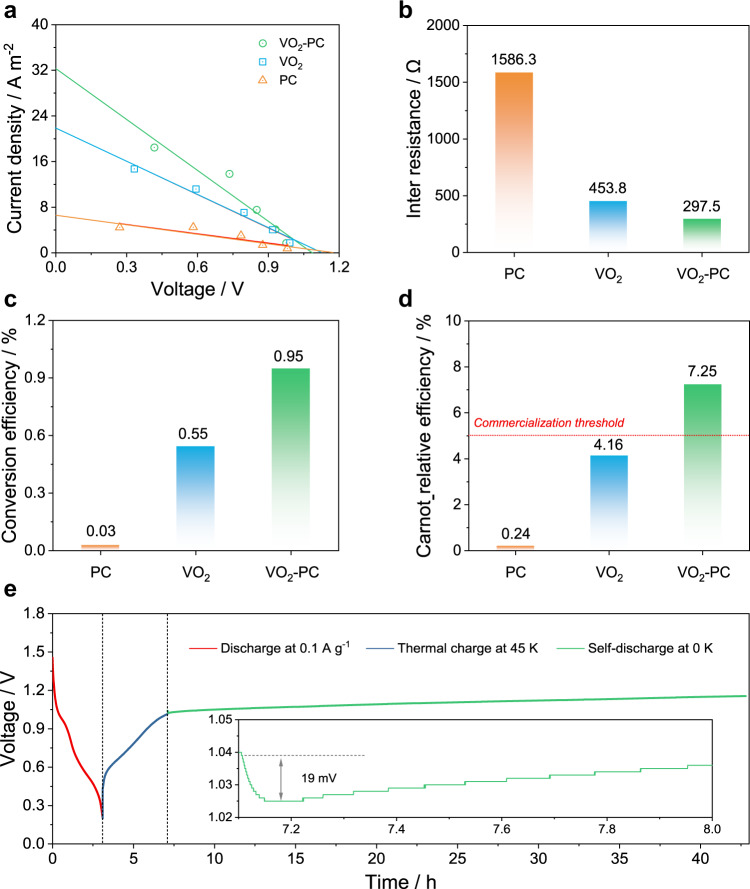
Heat-to-electricity conversion of ZTCCs. **a** Current–voltage plot, **b** inter resistance, **c** conversion efficiency, and **d** Carnot-relative efficiency for PC, VO_2_, and VO_2_-PC, respectively. **e** Self-discharging curve after eliminating the temperature gradient.

The rate behavior of the thermal charging cell was recorded by the galvanostatic discharge curves after charging with various temperature differences. As profiled in Supplementary Fig. 7a, the discharge time gradually increases from 240 to 3895 s with the input of temperature gradient from 5 to 45 K, indicating the conversion capability of ZTCCs from heat to electricity. Moreover, we have carried out the discharge performances of devices after charging with heat process and hybrid process to distinguish the ratio of heat charge part (Supplementary Fig. 7b). The VO_2_-PC-based ZTCC can obtain a moderate voltage of ~1.01 V after charging with a temperature difference of 45 K. The output voltage can be greatly enhanced to 1.6 V by introducing the power charge process. According to the discharging curves, the capacity obtained by the thermal process accounts for **~**46.7% of the capacity obtained with the hybrid charging mode, suggesting great potential of as-proposed ZTCC in practical applications. Moreover, the short-circuit current and open-circuit voltage were recorded by the plot in Fig. 3a after charging under 45 K with various resistances. As profiled, the VO_2_-PC-based ZTCC exhibits a higher current response (32.3 A m^−2^) than that of PC (6.5 A m^−2^) and VO_2_ (21.8 A m^−2^), suggesting its lower inter resistance. Consequently, a relatively low resistance value of 297.5 Ω can be obtained by the VO_2_-PC-based ZTCC, while the value is 1586.3 and 453.8 Ω for PC and VO_2_, respectively. This satisfying result could be mainly caused by the synergistic effect by introducing carbon matrix into metallic species. Besides, the heat-to-current conversion efficiency (*η*) of the ZTCC is estimated by Eq. (4), which can be expressed as^22^:

4 $$\eta =\frac{{\alpha }^{2}\Delta T}{4\kappa }\cdot \frac{d}{A{R}_{{{{{{{\rm{Cell}}}}}}}}}$$

where *κ* is the thermal conductivity, *d* is the inter-electrode spacing, *A* is the cross-sectional area, and *R*_Cell_ is the inter resistance of the ZTCCs. Based on the parameters summarized in Supplementary Table 1 and Supplementary Table 2, different energy conversion efficiency can be obtained, which still is a controversial point in thermoelectric. Details of efficiency calculation are shown in Supplementary Note 2. As plotted in Fig. 3c, the *η* of ZTCC reaches 0.95% by the VO_2_-PC-based ZTCC at 45 K, which is significantly higher than that of VO_2_ and PC-based ZTCCs. When considering the corresponded Carnot efficiency, the VO_2_-PC-based ZTCC can obtain the highest Carnot-relative efficiency (*η*_r_) of 7.25% at a Δ*T* of 45 K owing to its high power density (Fig. 3d). In addition, the *η*_r_ for VO_2_-PC-based ZTCC substantially surpasses the predicted commercialization threshold and some previously reported value^22,25,26^. In addition, the self-discharging curve is adopted to study the electrochemical behavior for ZTCC (Fig. 3e). After discharging at a current density of 0.1 A g^−1^ and thermal charge at a Δ*T* of 45 K, an output voltage of ~1.01 V can be rationally achieved. There is a negligible voltage drop (19 mV) occurred when eliminating the temperature gradient. It is worth mentioning that the gradual voltage growth following the self-discharge process with a temperature of 0 K can be attributed to the chemically self-charging behaviors^15^.

### Energy storage behavior of ZIBs

**Fig. 4:**
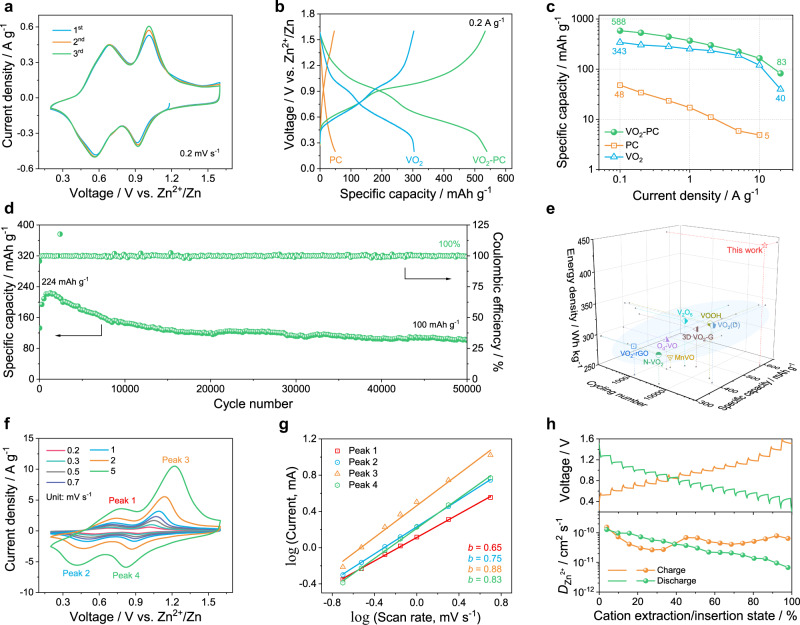
Electrochemical performance of ZIBs with various electrodes. **a** CV curves for the initial 3 cycles of VO_2_-PC at 0.2 mV s^−1^. **b** GCD curves, **c** Ragone plots, and **d** cycling stability at 10 A g^−1^. **e** Comparison of main electrochemical parameters of the VO_2_-PC-based ZIB with other reported devices. **f** CV curves at various scan rates. **g** The corresponding log (current) vs. log (scan rate) of each redox peak. **h** GITT curves and Zn^2+^ diffusion coefficient at charging/discharging.

To evaluate the energy storage ability of as-constructed devices, coin-type ZIBs are reasonably carried out. Figure 4a profiles the cyclic voltammetry (CV) curves of VO_2_-PC-based ZIB at a scan rate of 0.2 mV s^−1^. Clearly, two pairs of redox peaks at around 0.56/0.68 V and 0.92/1.01 V belong to the multiple insertion/extraction procedures of electrolyte ions in VO_2_ (Zn_*x*_VO_2_·*y*H_2_O ↔ VO_2_ + *x*Zn^2+^+ *y*H_2_O + 2*x*e^−^)^27^. The almost overlapping CV curves at the initial three cycles further indicate the highly reversible electrochemical behavior. Besides, the VO_2_-PC displays the largest enclosed CV area among PC and commercial VO_2_, suggesting its highest charge storage ability (Supplementary Fig. 8a). The galvanostatic charge/discharge (GCD) curves at a low current density of 0.2 A g^−1^ are in keeping with the same redox reactions of CV curves (Fig. 4b). As a result, a high discharge capacity of 539 mAh g^−1^ can be achieved for VO_2_-PC in this case, which is significantly higher than that of VO_2_ (303.8 mAh g^−1^) and PC (49.3 mAh g^−1^). Meanwhile, the highly reversible behavior and rate capability of VO_2_-PC are investigated by GCD tests at different current densities from 0.1 to 20 A g^−1^ (Supplementary Fig. 8b). Compared to the VO_2_ and PC, the VO_2_-PC exhibits an excellent capacitive performance together with high reversibility at each rate (Fig. 4c). Impressively, the reversible discharging capacity can still retain 80 mAh g^−1^ even at a high current density of 20 A g^−1^. When the current density was adjusted back to 0.1 A g^−1^, the specific capacity can be nearly recovered to 530 mAh g^−1^, which maintains about 91% of the initial discharging capacity. The corresponding GCD curves of the VO_2_-PC-based ZIB at various current densities are plotted in Supplementary Fig. 8c. The obvious electrochemical platforms that appeared in the charging and discharging curves directly confirm the mixed energy storage processes endowed by V-species. Ragone plots show the satisfying rate capability of VO_2_-PC. As summarized in Supplementary Fig. 8d, both a superior energy density of 442 Wh kg^−1^ at 112 W kg^−1^ and a high power density of 14.8 kW kg^−1^ at 43 Wh kg^−1^ can be reached based on the cathode mass, which surpasses those of the other devices with PC and VO_2_ as electrode materials. When taking into consideration of the total mass (${m}_{{{{\mbox{VO}}}}_{2}{{\mbox{-PC}}}}$ + ${m}_{{{\mbox{Zn}}}}$), a maximum energy/power density of 33.0 Wh kg^−1^/1105.4 W kg^−1^ can be retained, implying its satisfactory application as one of the systems for large-scale energy storage (Supplementary Note 3 and Supplementary Table 3). Remarkably, the specific capacity of VO_2_-PC-based ZIB still can retain approximately 100 mAh g^−1^ even over 50,000 cycles at a relatively high current density of 10 A g^−1^, and the corresponding Coulombic efficiency is about 100% for each cycle (Fig. 4d). Such results also are dramatically higher than that of PC- and VO_2_-based ZIBs (Supplementary Fig. 8e). Compared with the previously reported vanadium-based cathodes for aqueous ZIBs, the VO_2_-PC electrode exhibits outstanding performances in terms of specific capacity, cyclic stability, and energy density (Fig. 4e)^28–36^. These results demonstrate the satisfying ability of this combination of PC matrix and VO_2_ crystal in the storage of Zn^2+^ ion, which suggests that the VO_2_-PC would hold a promising performance to fulfill the requirements of Zn-based systems.

The rate performance of VO_2_-PC is greatly determined by its electrochemical kinetics, which is reasonably studied by CV measurements from 0.2 to 5 mV s^−1^ (Fig. 4f). Typically, the surface-induced capacitance and diffusion-controlled procedure can be indexed according to the relationship between peak current (*i*) and scan rate (*v*) as below: *i* = *av*^b^^37^. As known, *b* = 0.5 indicates that the electrochemical process is totally controlled by diffusion, while *b* = 1.0 represents the capacitance-dominated process. Notably, the calculated *b* values for peaks 1–4 are 0.65, 0.75, 0.88, and 0.83, respectively (Fig. 4g), which reveals that the electrochemical kinetics of VO_2_-PC is dominated by surface capacitive behavior. Furthermore, the capacity can be detailly divided into capacitance (*k*_1_*v*) and diffusion-controlled (*k*_2_*v*^1/2^) contribution by the following formula^38^:

5 $$i={i}_{{{{{{\rm{cap}}}}}}}+{i}_{{{{{{\rm{diff}}}}}}}={k}_{1}v+{k}_{2}{v}^{1/2}$$

As displayed in Supplementary Fig. 8f, a high capacitive contribution of 53% can be obtained at the scan rate of 0.2 mV s^−1^. With the increase of scan rate from 0.2 to 5 mV s^−1^, the capacitive contribution of VO_2_-PC electrode gradually increases from 53% to 87.5%, indicating fast charge transfer feature as well as good rate capability. In addition, the galvanostatic intermittent titration technique (GITT) was performed to investigate the Zn^2+^ diffusion behavior in the VO_2_-PC electrode during charging and discharging processes (Fig. 4h). The average diffusion coefficient of Zn^2+^ (*D*_Zn_) can be calculated as high as 10^−10.5^ cm^2^ s^−1^ for the whole charge and discharge, which suggests the fast transport of Zn^2+^ in the cathode. Such a high Zn^2+^ diffusion coefficient can correspond to the continuous PC network and embedded electroactive VO_2_. It is worth mentioning that the decrease of *D*_Zn_ value from about 10^−10^ to 10^−11^ cm^2^ s^−1^ in the discharge process can be attributed to the gradual increase of Zn^2+^ content in the VO_2_-PC electrode, which further indicates the good ability of VO_2_-PC cathode for ions diffusion.

### Energy storage mechanism of ZIBs

**Fig. 5:**
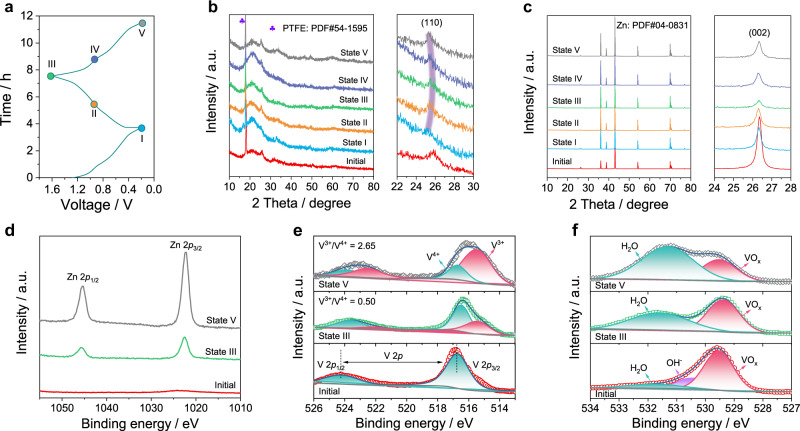
The mechanism of VO_2_-PC-based ZIBs. **a** The GCD curves at 0.1 A g^−1^. **b**, **c** The corresponding ex-situ XRD patterns of **b** VO_2_-PC cathode and **c** Zn-G anode. **d**–**f** The corresponding ex situ XPS spectra of **d** Zn 2*p*, **e** V 2*p*, and **f** O 1*s*.

Inspired by the superior performances of VO_2_-PC, various ex-situ measurements were conducted to reveal the detailed reaction mechanism during electrochemical tests. For convenience, the VO_2_-PC electrode at specific voltage used for analysis is marked as *state x*, as shown in Fig. 5a. Before the test, the VO_2_-PC-based ZIB is discharged to 0.2 V and then charged/discharged to the selected voltage state. From the XRD patterns of the VO_2_-PC cathode at various states (Fig. 5b), the characteristic peaks at ~18° and ~25° can be detected in all patterns, which could be assigned to the (100) plane of PTFE (PDF#54-1595) and the (110) plane of monoclinic VO_2_ (PDF#81-2392)^39^. During the charging process from state I to state III, the interlayer space corresponded to (110) plane is gradually reduced because of the slight positive shift of peaks at ~25°, which indicates the extraction of electrolyte ions from the VO_2_ crystal. Notably, the negative shift of (110) plane during the discharging process further confirms the insertion of electrolyte ions into VO_2_, suggesting a highly reversible behavior for ions storage. In addition, the byproduct-free Zn anode is also identified by the corresponding XRD patterns (Fig. 5c), meaning its highly reversible and durable feature. The added peak at ~26.3° can be well matched with the (002) plane of graphite, suggesting the successful modification of Zn surface^37^. Moreover, ex-situ XPS analyses were employed to explore the chemical states of the initial and the fully charged/discharged electrodes (Fig. 5d–f). In Fig. 5d, the high intensity of Zn 2*p* peaks in the state V clearly demonstrates the insertion of Zn^2+^ into VO_2_. The appearance of the Zn signal at state III is probably caused by the presence of residual zinc salts, indicating that most of Zn^2+^ can be extracted from the VO_2_-PC cathode^18^. To our knowledge, the GCD processes of V-based ZIBs could be accompanied by the valence change of V element in VO_2_ crystals. Compared with the V^4+^ existing in the initial electrode, the V 2*p* peaks move to low binding energy during discharge together with a high V^3+^/V^4+^ ratio of 2.65, which suggests the reduction of vanadium-oxide (Fig. 5e). In the charging process, the V^3+^/V^4+^ ratio decreases to 0.50, and the peaks of V 2*p* are nearly recovered to the initial state due to the oxidization of vanadium. Besides, the content of H_2_O gradually enhances during the discharging state and decreases during the charging state, which directly confirms that the solvation effect can promote the diffusion of H_2_O to VO_2_ along with the insertion of Zn^2+^ (Fig. 5f)^29^. The above results propose that the VO_2_-PC-based ZIBs exhibit a hybrid energy storage mechanism including the ions adsorption on PC and the redox reaction in VO_2_.

### Performance of wearable ZTCCs

**Fig. 6:**
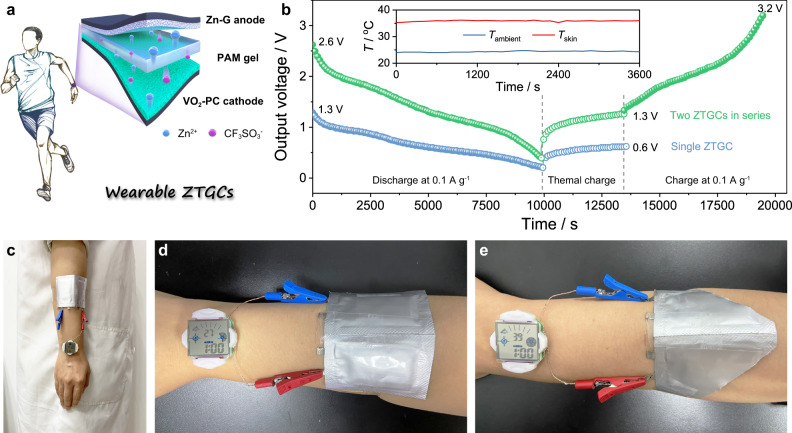
Proof of concept of ZTCCs at various modes. **a** Schematic drawing of the wearable ZTCCs. **b** Charge/discharge process at galvanostatic and/or thermal models. The inset shows the change of body heat and ambient temperature. **c**–**e** A smartwatch powered by two ZTCCs in series at **c**, **d** initial state and **e** the harsh condition.

Inspired by the satisfying performance of ZTCCs for energy storage and conversion, we further designed a quasi-solid-state device using polyacrylamide (PAM)-based gel for wearable application (Fig. 6a). Considering that the Zn^2+^ can be extracted from Zn_*x*_VO_2_·*y*H_2_O on the hot side and deposited on Zn electrode on the cold side, the VO_2_ cathode acts as an electrode for energy conversion and storage simultaneously. In such wearable ZTCCs, the Zn^2+^ can diffuse to VO_2_ firstly and react into Zn_*x*_VO_2_·*y*H_2_O after a fully discharged procedure. As profiled in Fig. 6b, the ZTCC can charge and discharge under multiple modes. When the ZTCC is thermally charged with the temperature difference between skin temperature (*T*_skin_) and ambient temperature (*T*_ambient_), the output voltage can slowly reach to ~0.6 V (Fig. 6b). It is worth mentioning that almost 1.3 V can be achieved with two ZTCCs in series (Δ*T* of ~12 K). Such integrated devices can be further galvanostatically charged from thermal charge state (~1.3 V) to fully charged state (3.2 V). To demonstrate the practical application of wearable ZTCCs in real conditions via a facile way, only two pouch ZTCCs connected in series were employed to power a smartwatch (Fig. 6c–e), which is much easier than that of practical e-TE or i-TE integration. Moreover, as-constructed ZTCCs can work normally even under harsh conditions (Fig. 6e), confirming the availability and durability of developed ZTCCs.

## Discussion

In summary, we have demonstrated the design and construction of ZTCCs with Zn anode and VO_2_-PC cathode, which is prepared by a universal solution-based strategy, for high-performance low-grade heat conversion and Zn^2+^ storage. It is worth mentioning that the evenly distributed VO_2_ in the PC provides abundant electroactive sites, delivering high capability and fast kinetics for Zn^2+^ storage. The carbon matrix acts as continuous pathways for charge transport and channels for electrolyte ions diffusion. Consequently, a high thermopower of 12.5 mV K^−1^ can be achieved by VO_2_-PC, which is beneficial to the synergistic effect of the thermogalvanic effect and thermodiffusion. As a proof-of-concept, ZTCCs exhibits a high output voltage of ~1 V and a record-breaking output power of 1220 μW as well as high Carnot-relative efficiency of 7.25% under the temperature difference of 45 K. Moreover, the VO_2_-PC exhibits an excellent Zn-ion storage behavior, such as a high specific capacity (588 mAh g^−1^ at 0.1 A g^−1^), good rate capability (80 mAh g^−1^ even at 20 A g^−1^), and impressive cycling stability over 50,000 cycles. Moreover, two wearable ZTCCs connected in series demonstrate high availability and durability in the electronics area. All the findings have confirmed that the proposed ZTCC would be a potential candidate for promising energy conversion and storage, and the detailed studies of involved mechanisms may provide deep insight for the development of zinc-based devices.

## Methods

### Preparation of VO_2_-PC

Typically, 1.0 g of ammonium metavanadate and 0.25 g dopamine hydrochloride were dissolved in 100 mL deionized water. Then, 200 mL of ethanol was added to the above solution with stirring for about 10 min. After that, 1.5 mL of NH_3_·H_2_O was dropped into it and stirred for another 2 h to obtain V-PDA precursors. Finally, the VO_2_-PC was prepared by the pyrolysis of V-PDA at 500 °C for 3 h with a heating rate of 3 °C min^−1^ under argon flow. In addition, the Mo_4_O_8_-PC and W_3_O_9_-PC were synthesized by the same strategy as above, only changing the added metal source to ammonium molybdate tetrahydrate and ammonium metatungstate.

### Electrochemical measurements

All the electrochemical performances for the conversion of low-grade heat to electricity were evaluated on a standard electrochemical workstation (CHI 660C) with a non-isothermal H cell using 0.5 mol L^−1^ Zn(CF_3_SO_3_)_2_. Typically, the working electrode was prepared by the coating of as-obtained samples onto graphite paper together with acetylene and polyvinylidene fluoride according to a mass ratio of 7:2:1. It is worth mentioning that the mass loading in the working electrode was about 1.2 mg cm^−2^. Besides, the Zn anode used was modified by graphite following pencil drawing^37^. For ZIB tests, the CV curves and GITT were performed by the Biologic VMP-300 workstation. The GCD measurements and cycling stability were collected on the CT3001A Land Battery Test System. Notably, the specific capacity and energy density of ZIBs were recorded directly from Land Battery Test System.

The quasi-solid-state ZTCCs were constructed by sandwiching the Zn-G anode, gel electrolyte, and VO_2_-PC cathode. The gel electrolyte was prepared through polymerization of acrylamide (AM) and subsequent electrolyte immersion. Briefly, 2.5 g of AM, 1.5 mg of *N*,*N*′-methylenebisacrylamide, and 10 μL of *N*,*N*,*N*′, *N*′-tetramethylethylenediamine were added into 10 mL of deionized water part by part with vigorous stirring at ~0 °C. After that, 0.25 g of potassium persulfate was dispersed into the above solution. When polymerizing with an ultraviolet lamp (60 W) for about 20 min, a transparent hydrogel was prepared. Finally, such hydrogel was immersed in an electrolyte solution to fabricate a flexible Zn(CF_3_SO_3_)_2_-PAM gel electrolyte.

### Material characterizations

SEM and TEM were employed on Hitachi S-4800 and JEOL JEM-2100, respectively. Powder XRD was obtained by a PANalytical Empyrean diffractometer with Cu Kα radiation (*λ* = 1.5406 Å). XPS was carried out by the KRATOS AXIS SUPRA instrument. Raman spectroscopy was conducted by the Horiba Scientific LabRAM HR.

## Supplementary information

Supplementary Information

## Data Availability

All relevant data that support the findings of this study are presented in the manuscript and supplementary information file. Source data are available from the corresponding author upon reasonable request.
