# Supplementary material for: Zinc ion thermal charging cell for low-grade heat conversion and energy storage
Source: Nat Commun. 2022 Jan 10;13:132. doi: 10.1038/s41467-021-27755-x (PMC8748914; doi:10.1038/s41467-021-27755-x)
Supplement: Supplementary file 1 — Supplementary Information [file 41467_2021_27755_MOESM1_ESM.pdf]

Supplementary Information

Zinc Ion Thermal Charging Cell for Low-Grade Heat Conversion and Energy Storage

Zhiwei Li,[1] Yinghong Xu,[1] Langyuan Wu,[1] Yufeng An,[1] Yao Sun,[1] Tingting Meng,[2] Hui Dou,[1] Yimin Xuan,[2*] Xiaogang Zhang[1*]

[1]Jiangsu Key Laboratory of Electrochemical Energy Storage Technologies, College of Material Science and Engineering, Nanjing University of Aeronautics and Astronautics, Nanjing 211106, China

[2]School of Energy and Power Engineering, Nanjing University of Aeronautics and Astronautics, Nanjing 210016, P. R. China

[*]Correspondence to X. Z. (azhangxg@nuaa.edu.cn) and Y. X. (ymxuan@nuaa.edu.cn)

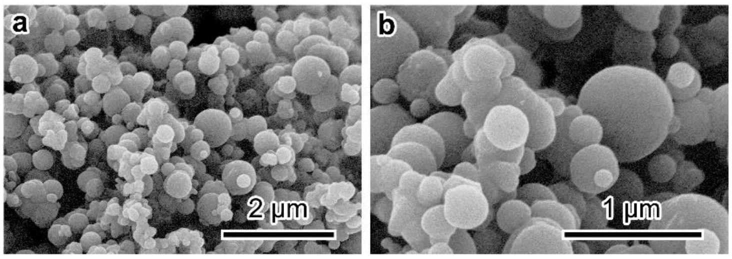

Supplementary Figure 1 SEM image of PC with **a** low and **b** high magnification.

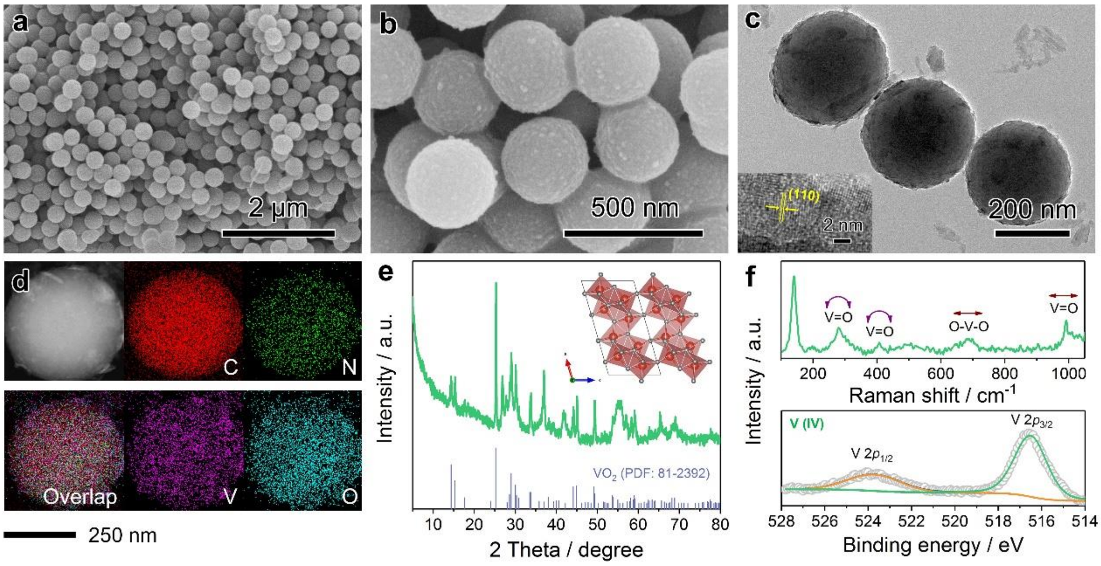

Supplementary Figure 2 Morphology and structural characterization of the VO_2-PC. **a,b** SEM and **c** TEM images. **d** EDX mapping images. **e** XRD pattern and the crystal structure of the VO_2-PC. **f** Raman spectrum and high-resolution XPS spectrum of V 2*p*.

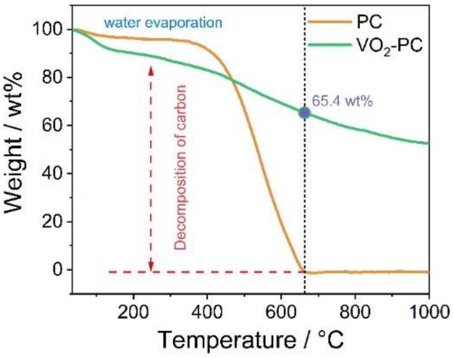

Supplementary Figure 3 TGA curves of PC and VO_2-PC.

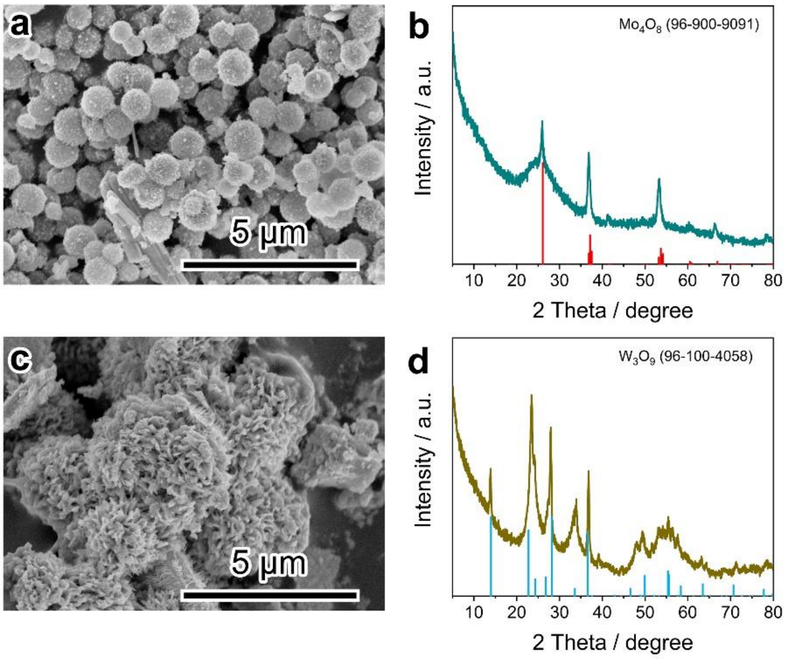

Supplementary Figure 4 The SEM image and XRD pattern for **a,b** Mo_4O_8 and **c,d** W_3O_9, respectively.

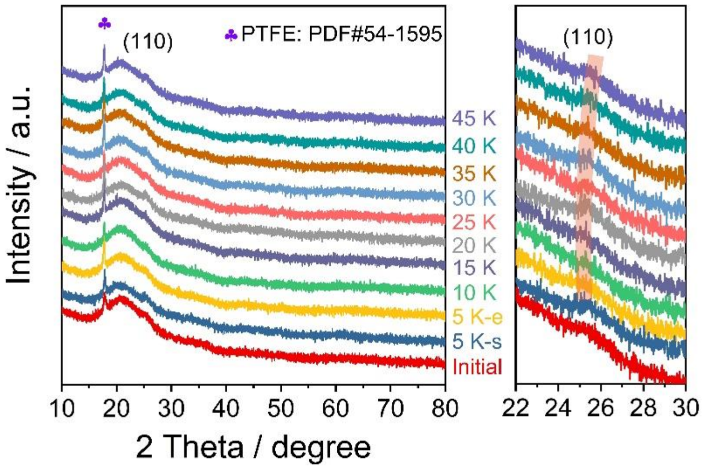

Supplementary Figure 5 *Ex-situ* XRD patterns of VO_2-PC cathode under various thermally charging states.

Supplementary Note 1: Relative contribution to the total thermopower of ZTCCs

The total thermal power achieved by our zinc ion thermal charging cells (ZTCCs) can be mainly delivered by the thermodiffusion and thermoextraction in a relatively complex system containing Zn^{2+}, $CF_3SO_3^-$, water, porous carbon and VO_2. Based on this consideration, the thermopower can be written as: $S = \alpha_{\mathrm{Zn/Zn^{2+}}} + S_{\mathrm{td(electrolyte)}} + S_{\mathrm{td(electrode)}} + \alpha_{ZnVO_2/VO_2}$, where $\alpha_{\mathrm{Zn/Zn^{2+}}}$ and α_{ZnVO_2/VO_2} is the contribution to thermopower from the redox reaction of $Zn^{2+} + e^- \leftrightarrow Zn$ and $Zn_xVO_2 \cdot yH_2O \leftrightarrow VO_2 + xZn^{2+} + yH_2O + 2xe^-$, respectively, S_{td} is the thermopower obtained from the thermodiffusion of electrolyte ions in both electrolyte and electrode. Therefore, we have carried out four cells (Pt|$Zn(CF_3SO_3)_2$|Pt, Zn|$Zn(CF_3SO_3)_2$|Zn, VO_2-PC|$Zn(CF_3SO_3)_2$|VO_2-PC, Zn_xVO_2-PC|$Zn(CF_3SO_3)_2$|Zn_xVO_2-PC) to determine the individual contribution of each effect. As summarized in Supplementary Figure 6a, the thermal charging response of Pt|$Zn(CF_3SO_3)_2$|Pt (0.99 mV K^{-1}) represents the thermodiffusion of Zn^{2+} and $CF_3SO_3^-$ in bulk electrolyte. Notably, when using Zn as electrodes, the value is 0.52 mV K^{-1}. This result suggests that the absolute value of thermopower for Zn/Zn^{2+} is 0.47 mV K^{-1}. Meanwhile, the thermopower from the reaction between Zn_xVO_2-PC and VO_2-PC is about 3.9 mV K^{-1}, distinguished by the values obtained by VO_2-PC|$Zn(CF_3SO_3)_2$|VO_2-PC and Zn_xVO_2-PC|$Zn(CF_3SO_3)_2$|Zn_xVO_2-PC. Due to the total thermopower of zinc ion thermal charging cell is 12.5 mV K^{-1}, relative contribution to the total thermopower can be determined as follows: 3.6% of redox entropy of Zn/Zn^{2+}, 30.1% of redox entropy of Zn_xVO_2/VO_2, 7.6% contribution of thermodiffusion of $Zn(CF_3SO_3)_2$ electrolyte, and 58.7% contribution of thermodiffusion of electrolyte ions in VO_2-PC electrode (Supplementary Figure 6b).

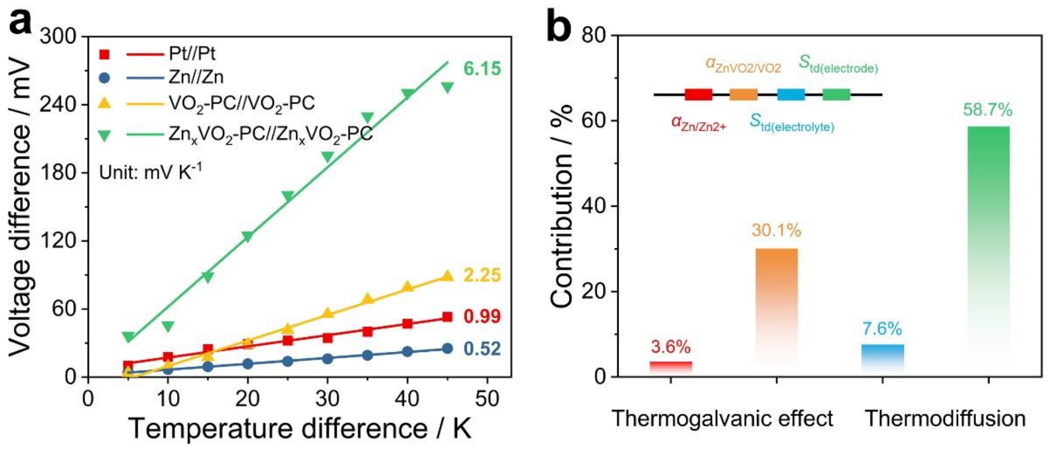

Supplementary Figure 6 **a** Open-circuit voltage *vs*. temperature difference for four cells. **b** Fractional contribution to thermopower of ZTCCs.

Supplementary Note 2: Calculation of conversion efficiency of ZTCCs

As reported, there are two kinds of approaches to calculate the conversion efficiency from low-grade heat to electricity by thermal devices. However, some significant differences should be noted.

Type I:

According to the literature (*Nat. Commun. 2014, 5, 3942*), the heat-to-current efficiency (η) for thermal devices is determined by the ratio of output electrical energy (W) to input thermal energy (Q). Here, the thermal energy includes the heat adsorbed (Q_H) for the heating process and the continuous energy input (Q_{dis}) at T_H. Therefore, the efficiency can be expressed as

$$\eta = \frac{W}{Q_H + Q_{dis}} \quad \text{(S1)}$$

In our work, when the zinc ion thermal charging cell is heated up from T_C to T_H, the heat consumption involved in the setup can be calculated by using

$$Q_H = (1 - \eta_{HX}) \sum m_i c_{p,i} \Delta T \quad \text{(S2)}$$

where m_i and $c_{p,i}$ represent the mass loading and specific heat capacity of the component i (like Zn, VO_2-PC, graphite, and 0.5 mol L^{-1} $Zn(CF_3SO_3)_2$ electrolyte). η_{HX} is the efficiency of heat recuperation. ΔT is the temperature difference applied, which can be determined by T_H-T_C.

Meanwhile, the energy consumption associated with chemical reactions requires continuous heat input to maintain isothermal conditions during discharge states, which can be defined as:

$$Q_{dis} = T_H \Delta S_H \quad \text{(S3)}$$

here, ΔS_H is the total entropy change, which equals to the entropy change at cathode (ΔS_{H+}) and anode (ΔS_{H-}).

Based on the partial molar entropy change (Δs_i) and the amount of component (i), the ΔS_H can be expressed as:

$$\Delta S_H = \int \Delta s_i dn_i \quad \text{(S4)}$$

For electrochemical reactions,

$$\frac{d\Delta G}{d\Delta T} = -\Delta S \tag{S5}$$

$$\Delta G = -nFE \tag{S6}$$

Therefore,

$$\Delta S_H = \int nF\frac{\Delta E}{\Delta T}dn_i = \alpha\int nFdn_i = \alpha q_{dis} \tag{S7}$$

where q_{dis} is the chargers transferred during discharge.

When applying equation S7 to S3,

$$Q_{dis} = T_H\alpha q_{dis} \tag{S8}$$

It is worth mentioning that the specific capacitance C (F g^{-1}) according to the report from F. Béguin's group for this non-linear GCD process:

$$C = \frac{2\times I\times S}{m\times\Delta V^2} = \frac{2\times I\int_{t_0}^{t_1}V(t)dt}{m\times\Delta V^2} \tag{S9}$$

where S (Vs) is the integral area of discharge curve. I (A), m (g), ΔV (V), and t (s) represent the current, the mass of active materials, the voltage range operated, and discharging time. After unit conversion and normalization of the active mass loading, the q_{dis} can be calculated.

Besides, the total net work output in this experiment is measured by galvanostatic discharge technique and can be calculated from:

$$W = \int_{t0}^{t1}(V_1 - V_0)I_{dis}dt \tag{S10}$$

Under above equations, we can obtain the heat-to-current efficiency of as-constructed zinc ion thermal charging cell.

$$\eta = \frac{\int_{t0}^{t1}(V_1-V_0)I_{dis}dt}{(1-\eta_{HX})\sum m_i c_{p,i}\Delta T + T_H\alpha q_{dis}} \tag{S11}$$

There is no heat recuperation during the experiments, so $\eta_{HX} = 0$.

As summarized, the conversion efficiency of as-constructed ZTCC can be calculated as high as 1.49 (11.36% of Carnot-relative efficiency), which is very high among current reported values.

Supplementary Table 1. Parameters for the efficiency calculation at various temperature differences.

	5 K	10 K	15 K	20 K	25 K	30 K	35 K	40 K	45 K
T_L (ºC)	25	25	25	25	25	25	25	25	25
T_H (ºC)	30	35	40	45	50	55	60	65	70
η_{Carnot} (%)	1.65	3.25	4.79	6.29	7.74	9.14	10.51	11.82	13.12
W (J)	0.004	0.007	0.013	0.023	0.039	0.056	0.076	0.112	0.376
q_{dis} (C)	0.022	0.034	0.060	0.095	0.148	0.190	0.228	0.302	0.929
α (mV K^{-1})	12.5	12.5	12.5	12.5	12.5	12.5	12.5	12.5	12.5
m_{Zn} (mg)	14.9	14.9	14.9	14.9	14.9	14.9	14.9	14.9	14.9
$c_{p,Zn}$ (J g^{-1} K^{-1})	0.316	0.316	0.316	0.316	0.316	0.316	0.316	0.316	0.316
$m_{VO2\text{-}PC}$ (mg)	2	2	2	2	2	2	2	2	2
$c_{p,VO2\text{-}PC}$ (J g^{-1} K^{-1})	1.725	1.725	1.725	1.725	1.725	1.725	1.725	1.725	1.725
$m_{Graphite}$ (mg)	23.2	23.2	23.2	23.2	23.2	23.2	23.2	23.2	23.2
$c_{p,Graphite}$ (J g^{-1} K^{-1})	0.641	0.641	0.641	0.641	0.641	0.641	0.641	0.641	0.641
$m_{electrolyte}$ (mg)*	0.269	0.416	0.734	1.162	1.810	2.323	2.788	3.693	11.36
$c_{p,electrolyte}$ (J g^{-1} K^{-1})	3.414	3.414	3.414	3.414	3.414	3.414	3.414	3.414	3.414
Q_H (J)	7.26	7.53	8.00	8.59	9.44	10.16	10.84	12.05	21.21
Q_{dis} (J)	0.08	0.13	0.23	0.38	0.60	0.78	0.95	1.28	3.98
η (%)	0.05	0.09	0.16	0.26	0.39	0.51	0.64	0.84	1.49
η/η_{Carnot} (%)	3.03	2.77	3.34	4.13	5.04	5.58	6.09	7.11	11.36
*$m_{electrolyte} = \rho \times V = \rho \times q_{dis}/(n \times 0.5\ M \times F) = 12.23 \times q_{dis}$ (mg)									

It is worth mentioning that the energy conversion efficiency calculated by this method is inappropriate for the ZTCC due to its adiabatic condition. Besides, such high

value could mainly be caused by the accuracy of parameters. As used, the specific heat capacity was typically measured by differential scanning calorimetry (DSC), which cannot rule out the existence of measurement errors. Thus, we should use another way (Type II) to evaluate the conversion efficiency of our ZTCC.

Type II:

By this way, the electrochemical conversion efficiency can be calculated by the equation:

$$\eta = \frac{V_{OC} I_{SC}}{4A\kappa(\Delta T/d)} \quad \text{(S12)}$$

When applying the relations ($V_{OC} = \alpha\Delta T$, $I_{SC} = \alpha\Delta T/R_{Cell}$), the heat-to-current efficiency can be expressed as

$$\eta = \frac{\alpha^2 \Delta T}{4\kappa} \cdot \frac{d}{AR_{Cell}} \quad \text{(S13)}$$

where α is the Seebeck coefficient, ΔT is the temperature difference, κ is the thermal conductivity, d is the inter-electrode spacing, A is the cross sectional area, and R_{Cell} is the inter resistance of the cell.

Supplementary Table 2. Parameters for the efficiency calculation of VO_2-PC based ZTCC at 45 K.

Seebeck coefficient (mV K^{-1})	12.5
Hot side temperature (K)	343.15
Cold side temperature (K)	298.15
Thermal conductivity (W m^{-1} K^{-1})	0.55
Cross sectional area of the cell (m^2)	0.000113
Inter-electrode spacing (m)	0.1
Inter resistance obtained by voltage-current plot (Ω)	297.5

As summarized in Supplementary Table 1, the energy conversion efficiency still can be calculated as 0.95% (7.25% of Carnot efficiency). Overall, the relatively high energy conversion efficiency can be mainly attributed to the unique energy conversion mechanism of ZTCCs. In fact, the ZTCC proposed in this work is a definite new

progress beyond typical thermoelectrics in the thermal electrochemical devices, which provides a new strategy to construct thermoelectrics with high performances. Moreover, the thermal charging cells with ultrahigh thermopower can be reasonably constructed by employing functional electrolyte with large size difference between cations and anions like organic electrolyte to replace common design of electrode as well as the introduction of near-surface reactions.

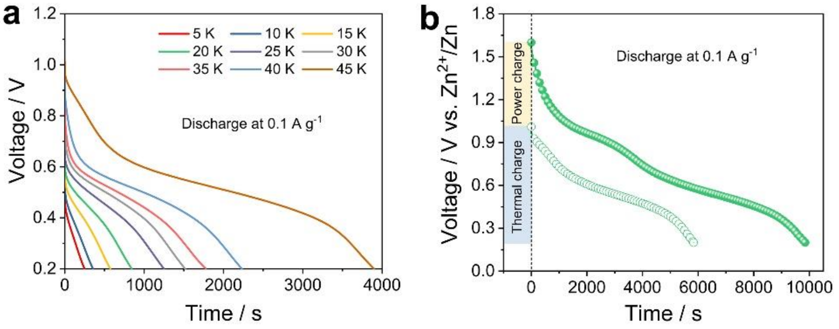

Supplementary Figure 7 a Galvanostatic discharge curves at 0.1 A g^{-1} after thermal charging at various temperature differences. **b** The discharge curves recorded after thermal charge and hybrid charge.

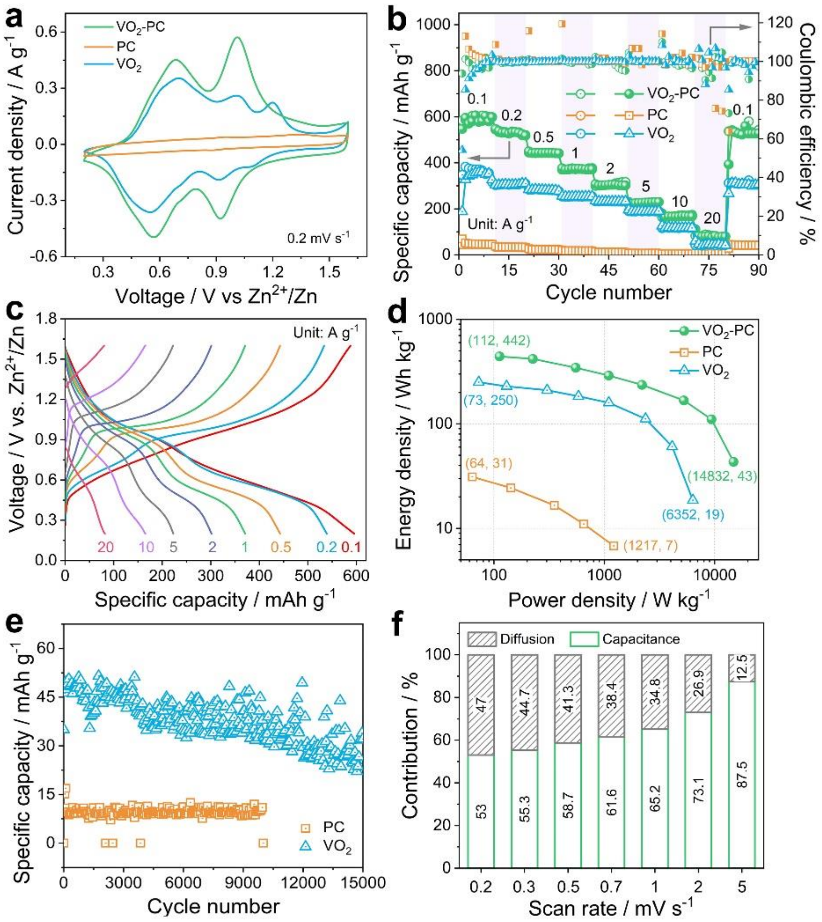

Supplementary Figure 8 a CV curves at 0.2 mV s^{-1} and **b** rate performance of VO_2-PC, VO_2, and PC. **c** GCD curves of VO2-PC at various current densities. **d** Ragone plots. **e** Cycling stability at 10 A g^{-1}. **f** Capacitive contributions at different scan rates.

Supplementary Note 3: Calculation of energy density for ZIBs.

It is worth mentioning that the zinc anode (diameter: 1.2 cm) used in our work is 25 μm in thickness, which means an areal mass of 13.2 mg cm^{-2}. Thus, we have provided such related results according to the total mass ($m_{VO2\text{-}PC}$+m_{Zn}). Consequently, a maximum energy/power density of 33.0 Wh kg^{-1}/1105.4 W kg^{-1} can be obtained, which implies its satisfactory application as one of systems for large-scale energy storage (Supplementary Table 2). In our opinion, the development of lightweight Zn electrode even metal-free anode is one of important strategies to construct ZIBs with high performance as well as promising applications.

Supplementary Table 3. Energy density and power density for VO_2-PC//Zn-G.

Current density (A g^{-1})	Based on cathode mass (1.2 mg cm^{-2})		Based on total mass (16.1 mg cm^{-2})	
	E (Wh kg^{-1})	*P* (W kg^{-1})	*E* (Wh kg^{-1})	*P* (W kg^{-1})
0.1	442	112.3	33.0	8.3
0.2	418.3	224.9	31.2	16.8
0.5	344.6	551.3	25.7	41.0
1	289.8	1096.8	21.6	81.7
2	236.2	2196.9	17.6	163.8
5	167.4	5262.8	12.5	392.2
10	110.5	9364.4	8.2	698.0
20	43.3	14831.6	3.2	1105.4
